# A bioavailable cathepsin S nitrile inhibitor abrogates tumor development

**DOI:** 10.1186/s12943-016-0513-7

**Published:** 2016-04-21

**Authors:** Richard D. A. Wilkinson, Andrew Young, Roberta E. Burden, Rich Williams, Christopher J. Scott

**Affiliations:** Molecular Therapeutics, School of Pharmacy, Queen’s University Belfast, 97 Lisburn Road, Belfast, BT9 7BL United Kingdom; Centre for Cancer Research and Cell Biology, Queen’s University Belfast, 97 Lisburn Road, Belfast, BT9 7BL United Kingdom

**Keywords:** Cathepsin, Inhibitor, Cancer, Tool, Therapeutic

## Abstract

**Background:**

Cathepsin S has been implicated in a variety of malignancies with genetic ablation studies demonstrating a key role in tumor invasion and neo-angiogenesis. Thus, the application of cathepsin S inhibitors may have clinical utility in the treatment of cancer. In this investigation, we applied a cell-permeable dipeptidyl nitrile inhibitor of cathepsin S, originally developed to target cathepsin S in inflammatory diseases, in both in vitro and in vivo tumor models.

**Methods:**

Validation of cathepsin S selectivity was carried out by assaying fluorogenic substrate turnover using recombinant cathepsin protease. Complete kinetic analysis was carried out and true *K*_i_ values calculated. Abrogation of tumour invasion using murine MC38 and human MCF7 cell lines were carried out in vitro using a transwell migration assay. Effect on endothelial tube formation was evaluated using primary HUVEC cells. The effect of inhibitor in vivo on MC38 and MCF7 tumor progression was evaluated using cells propagated in C57BL/6 and BALB/c mice respectively. Subsequent immunohistochemical staining of proliferation (Ki67) and apoptosis (TUNEL) was carried out on MCF7 tumors.

**Results:**

We confirmed that this inhibitor was able to selectively target cathepsin S over family members K, V, L and B. The inhibitor also significantly reduced MC38 and MCF7 cell invasion and furthermore, significantly reduced HUVEC endothelial tubule formation in vitro. In vivo analysis revealed that the compound could significantly reduce tumor volume in murine MC38 syngeneic and MCF7 xenograft models. Immunohistochemical analysis of MCF7 tumors revealed cathepsin S inhibitor treatment significantly reduced proliferation and increased apoptosis.

**Conclusions:**

In summary, these results highlight the characterisation of this nitrile cathepsin S inhibitor using in vitro and in vivo tumor models, presenting a compound which may be used to further dissect the role of cathepsin S in cancer progression and may hold therapeutic potential.

**Electronic supplementary material:**

The online version of this article (doi:10.1186/s12943-016-0513-7) contains supplementary material, which is available to authorized users.

## Background

Proteases are involved in a variety of physiological processes wherein they facilitate the irreversible hydrolysis of substrate proteins [1]. The scope of protease activity provided by the genome is reflected by the identification of more than 500 known protease genes [2, 3]. Unsurprisingly, due to the role proteases play in areas such as signalling, receptor activation and chemokine/cytokine processing, dysregulated proteases are frequently involved in pathological conditions via various mechanisms [4–6]. As a result, there has been a surge in the development of inhibitors to further elucidate the role of proteases in disease [7–10].

Cathepsin S (CTSS) is a lysosomal protease which has been shown to be expressed in a number of inflammatory conditions including autoimmune, cardiovascular disease and cancer [11, 12]. In cancer, elevated CTSS expression has been associated with poorer outcome in grade IV astrocytoma and colorectal carcinomas [13, 14]. Furthermore, genetic ablation of CTSS in murine models resulted in diminished tumorigenicity [15, 16]. CTSS has been shown to cleave a variety of substrates including extracellular matrix (ECM) proteins, pro- and anti-angiogenic peptides and junctional adhesion proteins indicating a role in invasion, angiogenesis and metastasis [17–19].

Increased CTSS expression at the tumor site has been reported to be a result of tumor associated macrophage (TAM) recruitment, with the presence of CTSS dependent on IL-4, indicative of an M2 phenotype [20–22]. In addition to TAMs, tumor cells and recruited stromal cells can contribute CTSS to the microenvironment, highlighted through clinical observations [13] and investigations using stratified murine tumor gene depletion models [16, 19].

Previously, we have focussed our attention on developing and assessing inhibitors that can inhibit CTSS protein that is secreted into the tumor microenvironment, using protein based inhibitors such as a propeptide fusion protein [23] as well as an antibody inhibitor, FSN0503, which displays high selectivity towards CTSS [24]. However, our recent findings that CTSS may be able to promote pro-tumorigenic signalling through lysosomal cleavage of CD74 (invariant chain) to modulate CCL2 expression [6], highlights that an inhibitor that can target both extra- and intracellular activities may also have utility. To investigate this, we have now synthesised a small molecule inhibitor that was originally developed by Merck-Frosst [25]. This dipeptidyl nitrile inhibitor (Compound 6) was shown to possess 225-fold selectivity toward CTSS over cathepsin K, and a more than 560-fold selectivity against cathepsin L and cathepsin B [26]. Furthermore, it has previously been utilised in vivo studying lung granulomas and atherosclerotic lesions, demonstrating cell penetration and cross species reactivity [27, 28].

This investigation describes the application of this compound for the first time in tumor models. Previous studies have validated IC_50_ values for compound 6 versus CTSS, K, L and B. Here, we profile true *K*_i_ values for the inhibitor towards CTSS over the other related cathepsins confirming selectivity of compound 6. We also show that compound 6 can reduce block intracellular cleavage of known CTSS substrate lip10; attenuate colorectal and breast cell line invasion, and inhibit endothelial tube formation. Selectivity of the inhibitor to CTSS was confirmed using a cellular MC38 shRNA knock-down model. Finally, we demonstrate that compound 6 has therapeutic utility in murine models, blocking tumor progression using both a colorectal syngeneic and a breast xenograft tumor model.

## Methods

### Cell culture

Raji (human B cell) (ATCC), MC38 (murine colorectal carcinoma) (ATCC) and MCF7 (human breast adenocarcinoma) (ATCC) were cultured in media containing 10 % FCS (Sigma Aldrich, UK) and 50 U/mL Penicillin and 50 μg/ml streptomycin (Life Technologies, UK). HUVEC (Cellworks) primary cells were cultured in large vessel endothelial cell growth medium (Cellworks) containing human large vessel endothelial cell growth supplement (KC1016) and antibiotic supplement (KC1019). Cells were maintained in 5 % CO_2_ at 37 °C. MCF7 cell line was validated by STR profiling by LGC Standards in May 2011. All subsequent cells have only been taken from these validated stocks.

### Synthesis of compound 6

To a solution of Boc-Cys-OMe (0.88g, 3.74 mmol) in DMF (30 mL) at 0 °C was added K_2_CO_3_ (0.51 g, 3.74 mmol) and MeI (0.23 mL, 3.74 mmol) warmed to room temperature and stirred for 20 h. The reaction was diluted with a 9:1 water/NH_4_Cl (2 M aq. Solu.) and extracted with EtOAc (×2). The organic extracts were washed with 5 % brine, dried over Na_2_SO_4_ and concentrated. Dissolved in MeOH (10 mL) and added to a 2 N methanolic-HCl solution and stirred at room temperature for 3 h. The mixture was concentrated and the solid was washed with MTBE (50 mL ×3) to afford (S)-methyl 2-amino-3-(methylthio)propanoate hydrochloride) as a clear light yellow oil (0.62 g, 90 %). Dissolved in MeOH (8 mL) and cooled to -78 °C, added 2,2,2,4′-trifluoroacetophenone (0.60 g, 3.35 mmol) and MeOK (0.47 g, 6.70 mmol). The reaction was slowly warm to room temperature whilst stirring for 20 h – solution A. To a solution of NaBH_4_ (0.51 g, 13.4 mmol) in DME (10 mL) at 0 °C was added a 2 M solution of ZnCl_2_ in Et_2_O (8.03 mL). The mixture was allowed to age for 20 h –solution B. Solution A was cooled to -40 °C, diluted with MeCN (28 mL) and slowly added solution B over 20 min, then stirred at -40 °C for 2.5 h. The reaction was quenched with acetone (40 mL) over 20 min and then warmed to room temperature. The mixture was poured into an ice/water mix (250 mL), the pH was adjusted to 5 with 1 N HCl and extracted with EtOAc (200 mL ×3). The organic extracts were washed with brine (400 mL), dried over Na_2_SO_4_ and concentrated. Dissolved in DMF (15 mL), added 1-amino-1-cyclopropanecarbonitrile hydrochloride (0.39 g, 3.35 mmol), HBTU (1.59 g, 4.20 mmol) and NMM (0.54 mL, 4.00 mmol) and stirred at room temperature for 20 h. Diluted the reaction with water (120 mL) and extracted with EtOAc (100 mL ×2). The combined organic extracts were washed with 5 % brine (120 mL ×2) and brine (140 mL), dried over Na_2_SO_4_ and concentrated. The residue was purified by column chromatography (40 g column) using 0 to 75 % EtOAc in hexanes to afford (S)-*N*-(1-cyanocyclopropyl)-3-(methylthio)-2-(((S)-2,2,2-trifluoro-1-(4-fluorophenyl)ethyl)amino)propanoate as a clear colourless oil (0.36 g, 29 %). To a solution of (S)-*N*-(1-cyanocyclopropyl)-3-(methylthio)-2-(((S)-2,2,2-trifluoro-1-(4-fluorophenyl)ethyl)amino)propanoate (0.34 g, 0.91 mmol) in EtOAc (8.78 mL) was added NaTg•2H_2_O (3.56 mg, 0.01 mmol) and TBAH (15.6 mg, 0.05 mmol) followed by addition of H_2_O_2_ (30 % w/w in water, 0.23 mL). The reaction was stirred at room temperature for 3 h. Diluted the reaction mixture with EtOAc (70 mL) and washed with a solution of 2 M Na_2_S_2_O_3_ (100 mL ×2) and brine (100 mL), dried over Na_2_SO_4_ and concentrated to afford (S)-*N*-(1-cyanocyclopropyl)-3-(methylsulfonyl)-2-(((S)-2,2,2-trifluoro-1-(4-fluorophenyl)ethyl)amino) propanoate (compound 6) as a white powder (0.29 g, 79 %); ^1^H-NMR (400 MHz, *d*_*6*_-DMSO) δ 9.05 (s, 1H), 7.45 (dd, *J* = 8.0, 6.0 Hz, 2H), 7.25 (dd, *J* = 8.0, 6.0 Hz, 2H), 4.37 (quintet, *J* = 8.0 Hz, 1H), 3.74-3.66 (m, 1H), 3.54-3.46 (m, 2H), 3.17 (dd, *J* = 16.0, 4.0 Hz, 1H), 3.12 (s, 3H), 1.42-1.33 (m, 2H), 1.05-0.98 (m, 1H), 0.79-0.74 (m, 1H); LC-MS > 98 %, *m/z* = 408.12 [M + H].

### Inhibition of cathepsin activity using compound 6

Inhibition of cathepsin activity using compound 6 Recombinant cathepsin activity: Analysis of recombinant cathepsin activity was performed in a 96-well plate. All assays were performed in triplicate in the presence of sodium acetate assay buffer (sodium acetate 100 mM, EDTA 1 mM, Brij 0.1 %, and Dithiothreitol 2 mM, pH 5.5). Recombinant CTSS (4 nM), K (4.25 nM), V (4 nM), L (4 nM) and B (3.5 nM) (Calbiochem, UK) was incubated with compound 6 at a range of concentrations. The concentration of recombinant protein used in these assays was assumed to be equivalent to the active enzyme concentrations. Cathepsin activity was monitored using peptidyl fluorescent substrates; Cbz-VVR-AMC (20 μM, CTSS), Cbz-FR-AMC (20 μM, Cathepsins K, V and at 5 μM for cathepsin L) and Cbz-RR-AMC (20 μM, cathepsin B). Protease activity was monitored over the period of 1 h using a fluorometer (Flurostar Optima) with excitation at 390 nm and emission at 460 nm. Progress curve data points generated by compound 6 were fitted to equation 1 using GraphFit software.

1 $$ v={v}_o\ \frac{\left[E\right]-\left[I\right]-{K}_{i\left(\mathrm{app}\right)}+\sqrt{{\left(\left[E\right]-\left[I\right]-{K}_{i\ \left(\mathrm{app}\right)}\right)}^2+4\left[E\right].{K}_{i\ \left(\mathrm{app}\right)}}}{2\left[E\right]} $$

Where, $v$_o_ is the steady state rate, $E$ is the enzyme concentration and $I$ is the inhibitor concentration. Using this equation values of $v$ were generated for each concentration of inhibitor. Using these values, Morrison Plots were subsequently produced ($v$ versus [I]), allowing determination of *K*_i (app)_ values. To account for any completing substrate these values were corrected using equation 2, allowing the generation of true *K*_i_ values for compound 6 versus cathepsins S, K, V, L and B.

2 $$ {K}_i = {K}_{i\ \left(\mathrm{app}\right)}/\left(1+\frac{\left[S\right]}{K_m}\right) $$

*Cathepsin activity in lysates:* For analysis of CTSS-like activity in MC38 cell lysates, MC38 cells were grown to confluency, harvested and lysed on ice using sodium acetate lysis buffer (Sodium acetate 100 mM, sodium chloride 100mM, Triton X-100 0.1 %, pH 5.5). Lysates were quantified by BCA, before addition to black-bottom 96-well plate at 100 μg per well. The lysates were incubated in 100 mM phosphate buffer (pH 7.5) at 37 °C for 1 h to inactivate cathepsins B and L. Following this step, the lysates were incubated in MES buffer (MES 500 mM, EDTA 1mM, Dithiothreitol 2 mM, pH 5.5) to return the pH to 6, compound 6 added, and the lysates incubated for 30 min at 37 °C. The lysates were then evaluated for CTSS-like activity using 20 μM Cbz-VVR-AMC and fluorogenic substrate turnover monitored over the period of 1 h using a fluorometer (Flurostar Optima) as described above. The result was presented in triplicate, expressing relative fluorescent units versus time in minute ± SEM.

### Western blotting

Western blotting was carried out as previously described [29] using the following primary antibodies: mouse anti-human CD74 (1:400) (sc-47741, Santa Cruz, USA [30]), rat anti-mouse CD74 (1:1000) (555317, BD Biosciences, USA [31]) and rat anti-α-tubulin (1:10000) (ab6160, Abcam, UK [32]). The membrane was subsequently incubated with appropriate secondary antibody; goat anti-mouse HRP conjugate (1:10000) (172-1011, BioRad, UK [29]) or rabbit anti-rat HRP conjugate (1:40000) (ab102199, Abcam, UK [29]). Proteins were detected by chemiluminescence protocol and exposed using the BioRad Molecular Imager ChemiDoc XRS+ Imaging System (BioRad, USA).

### Invasion assays

A 24-well transwell plate (Corning, UK) containing 8.0 μm polycarbonate membrane was coated with Matrigel (1 mg/mL) (BD Biosciences). MC38 and MCF7 cells were seeded into the upper well at a density of 2.5x10^5^ per well in serum-free media in the presence of compound 6 at a range of concentrations and invasiveness evaluated as previously described [24]. Each condition was performed in duplicate, with 9 images taken per membrane at X20 magnification. Images were analysed using ImageJ and figures were generated using GraphPad Prism. Results demonstrate the mean number of cells per field of view per group ± SEM. Statistical analysis carried out by analysis of variance with Tukey test comparing all conditions.

### Endothelial tube formation assay

A 48-well plate was coated with Matrigel (10 mg/mL) and incubated at 37 °C for 1 h. 1×10^5^ HUVECs were seeded per well in triplicate, treated with compound 6 (100 nM/10000 nM) and incubated for 18 h. Imaging was performed using a Nikon Eclipse microscope with six images taken from triplicate wells and analysis carried out using ImageJ. Figures were generated using Graphpad Prism displaying the average total tubule length per field of view. The result was expressed as the mean total tubule length per group ± SD. Statistical analysis carried out by analysis of variance with Tukey test comparing all conditions.

### Lentiviral generation of stable shRNA lines

CTSS knock down construct sh835 was purchased from Sigma Aldrich, UK and were transduced into MC38 cells using lentiviral particles as previously described [6]. MC38 cells containing knockdown construct were continuously selected for using puromycin (6 μg/mL). Successful knock-down of CTSS was confirmed by RT-PCR.

### RT-PCR

RNA was extracted using STAT - 60 (Biogenesis, UK) according to manufacturer’s protocol and cDNA generated using Improm-II reverse transcriptase kit (Promega, UK) as previously described [29]. For amplification of the CTSS and GAPDH cDNA, the following primer sets were used: CTSS forward primer 5′ GGGATCTCTGGAAGAAAACCC’3 and reverse primer 3′- TTCGGAGACTGTCGGGGAAT’5. GAPDH forward primers 5′-AAGGTCATCCCAGAGCTGAA-3′ and reverse primer 3′CTGCTTCACCACCTTCTTGA-5′.

### In vivo evaluation of CD74 degradation in murine spleens

All mice used in these experiments were supplied with housing and subsequent experimentation was carried out in accordance with the Animal (Scientific Procedures) Act 1986, following UKCCCR guidelines and approved by the Ethical Review Committee with Queen’s University Belfast. 20 week old C57BL/6 mice were intraperitoneally injected with either compound 6 (100 mg/kg) or control. Spleens were harvested after 18 h and homogenized in RMPI1640 using nylon mesh. Red blood cells were lysed using ammonium chloride potassium (ACK) lysis buffer. Whole splenocytes were then lysed and protein analysed by western blot using rat anti-mouse CD74 antibody, as described previously.

### MC38 syngeneic model

Six to eight week old C57BL/6 mice were subcutaneously injected with 5.0×10^6^ MC38 cells resuspended in growth factor reduced matrigel (4 mg/ml, diluted in sterile PBS) into the right flank on day 0. Upon tumors reaching 100 mm^3^, mice were then treated with compound 6 (2 mg compound 6 in 4 % DMSO: 96 % peanut oil; 100 mg/kg) or vehicle control (4 % DMSO: 96 % peanut oil) via intraperitoneal injection every 2 to 3 days. Tumor volumes were calculated as Volume = length × breadth × *π*/6 (*n* = 5/group). Data was presented as mean tumor volume per group ± SEM. Statistical analysis carried out by student’s *t* test. Blood serums were sampled at day 13 from mice and subjected to CCL2 ELISA (R&D Systems, UK) as previously described [6].

### MCF7 xenograft model

Six to seven week old BALB/c nude mice were subcutaneously injected with an oestrogen pellet (approx 0.5 mg). 10 days later, mice were subcutaneously injected with 3.0x10^6^ MCF7 cells suspended in Matrigel (4 mg/ml, diluted in PBS). Upon tumors reaching 100 mm^3^, mice were then treated with compound 6 (2 mg compound 6 in 4 % DMSO: 96 % peanut oil; 100 mg/kg) or vehicle control (4 % DMSO: 96 % peanut oil) via intraperitoneal injection every 2 to 3 days. Tumor volume was calculated as described previously (*n* = 6/group). Data was presented as the mean tumor volume per group ± SEM. Statistical analysis carried out by student’s *t* test.

### Immunohistochemistry

MCF7 tumor sections (6 µm) were formalin fixed, paraffin-embedded and subjected to immuno-staining as previously described [16]. Sections (*n* = 4/group) were incubated in rabbit anti-human Ki67 primary antibody (1:300) (ab155580, Abcam, UK [16]). Slides were subsequently stained with biotinylated goat anti-rabbit (1:300) (BA-1000, Vector, UK [16]) secondary antibody. The number of Ki67 positive cells was assessed by random selection of 10 fields of view per tumor under X20 magnification using a Leica DM5500B microscope and AL software. The number of Ki67 positive cells was presented as the mean Ki67 positive cells ± SEM. Statistical analysis carried out by student’s *t* test.

### TUNEL staining

Paraffin-embedded MCF7 tumor sections (6 μm) were stained for apoptosis using terminal deoxynucleotidyl tranferase-mediated dUDP-nick-end labelling (TUNEL)-based TumorTACS™ In Situ Apoptosis Detection Kit (R&D Systems, UK [16]) as per the manufacturer’s instructions. TUNEL staining was assessed by random selection of 10 fields of view per tumor under a X20 magnification using a Leica DM5500B microscope and AL software. The number of TUNEL positive cells were counted and presented as the mean TUNEL positive cells ± SEM. Statistical analysis carried out by student’s *t* test.

### MTT cell viability assays

Effects on cell proliferation following compound 6 treatment or presence of knock-down construct sh835 were assessed by MTT assays as previously described [6]. Absorbance values were measured at 570 nM and results presented as mean ± SD.

## Results

### Evaluation of CTSS clinical candidate inhibitor

**Fig. 1:**
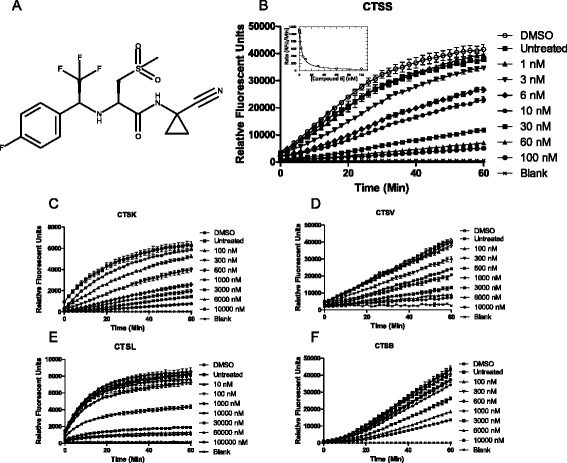
Compound 6 blocks recombinant CTSS in a concentration dependent manner. **a** Structure of compound 6. Inhibitory activity of compound 6 versus **b**) recombinant CTSS, inset: Morrison plot for calculation of *K*_i_ **c**-**f**) Inhibitory activity of compound 6 versus recombinant cathepsin K, V, L and B was measured by fluorescent peptidyl substrate assay

To assess the inhibition of both intra- and extracellular CTSS in our models we synthesized a dipeptide nitrile based inhibitor (compound 6), previously reported as a potent and selective inhibitor of human and murine CTSS (Fig. 1a) [26]. This compound has been previously used to investigate the therapeutic potential of CTSS in atherosclerosis [27]. The compound was synthesized in-house and its effectiveness against a panel of human cathepsin recombinant proteases was assessed.

Analysis of fluorimetric substrate turnover by CTSS demonstrated that compound 6 blocked CTSS activity in a concentration dependent manner (Fig. 1b). Furthermore compound 6 inhibited the activity of recombinant cathepsins K, V and B to varying degrees with limited activity against L (Fig. 1c-f). We then determined the *K*_i_ of compound 6 against each of these enzymes and subjected to non-linear analysis and fitted to Morrison kinetics [33]. This demonstrated that there was a high preference of compound 6 towards human recombinant CTSS (2.71 nM) over the other closely related proteases (CTSK 155 nM; CTSV 784 nM; CTSL 7870 nM; CTSB 1940 nM) (Additional file 1: Figure S1), in agreement with previously reported IC_50_ selectivities [26].

### Compound 6 blocks intracellular degradation of lip10

**Fig. 2:**
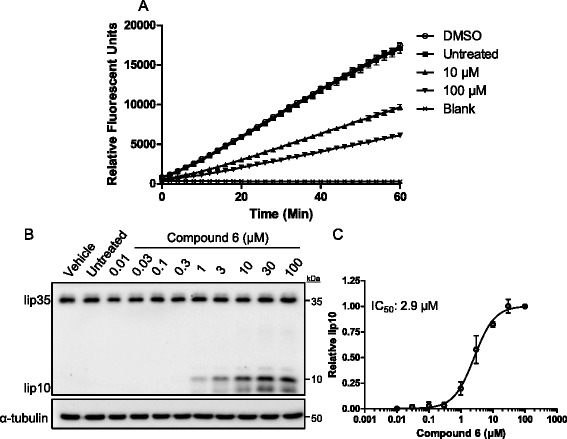
Compound 6 reduces MC38 CTSS-like activity and blocks intracellular degradation of CD74. **a** MC38 cells were lysed and treated with compound 6 demonstrating reduction of CTSS-like activity **b**) Compound 6 permeated the cell membrane of human Raji cells and blocked degradation of CD74 fragment lip10, a canonical endosomal CTSS substrate. **c** Evaluation of lip10 band intensity revealed an IC_50_ value of 2.9 µM for compound 6

To assess the effect of compound 6 on CTSS activity in a more complex milieu, we firstly incubated the inhibitor with murine MC38 colorectal carcinoma cell lysates, and measured turnover of a fluorogenic substrate Cbz-VVR-AMC. Addition of compound 6 to MC38 lysates demonstrated a concentration dependent reduction in CTSS-like activity (Fig. 2a).

Next we assessed the effectiveness of the inhibitor towards a physiologically relevant substrate. The invariant chain (CD74) is a known intracellular substrate of CTSS and when CTSS activity is ablated through inhibition or deletion, it leads to accumulation of a 10 kDa invariant chain fragment (lip10) [34, 35]. Thus, monitoring of this fragment in antigen presenting cells can inform the ability of CTSS inhibitors function within the cell. Raji cells were treated with compound 6 and the accumulation of the lip10 fragment was analysed by western blot (Fig. 2b). Semi-quantitative analysis of these bands by densitometry revealed compound 6 to have an IC_50_ of 2.9 μM (Fig. 2c).

### Inhibition of CTSS blocks tumor cell invasion and endothelial tube formation

**Fig. 3:**
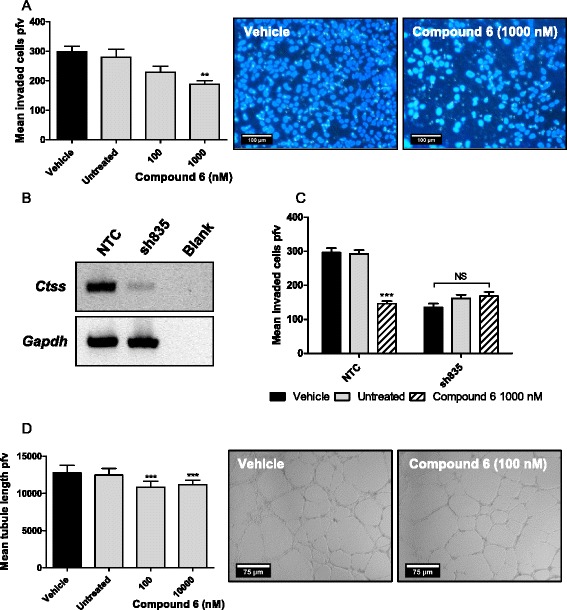
Compound 6 blocks CTSS mediated MC38 cell invasion and HUVEC endothelial tube formation. **a** Compound 6 attenuates MC38 invasion *Left*: Treatment of MC38 cells with 1000 nM compound 6 significantly reduced tumor cell invasion as determined by measuring invaded cells per field of view (pfv) (**P<0.01). *Right*: representative images. To evaluate compound 6 selectivity in a cellular assay, murine colorectal cell line MC38 was transfected with either a non-targeting construct (NTC) or CTSS knock-down construct (sh835). **b** Diminished CTSS expression was confirmed by RT-PCR. **c** The invasiveness of the MC38 NTC and sh835 cells was analysed by transwell assay. MC38 sh835 cells demonstrated reduced invasive potential compared to the MC38 NTC cells (***P<0.001). Treatment of MC38 NTC cells with compound 6 (1000 nM) resulted in a significant reduction in invasion (***P<0.001). No further effect was observed with treatment of MC38 sh835 cells with compound 6 (1000 nM). **d** *Left*: Compound 6 significantly reduced HUVEC endothelial tube formation approximately 10 % from 100 nM (***P<0.001). *Right*: representative images

Previously we have demonstrated the effect of blocking cell invasion through CTSS shRNA mediated depletion using MC38 cells [16]. Here we found that treatment with compound 6 resulted in a dose dependent inhibition of MC38 cell invasion (Fig. 3a). In parallel to these experiments, we assessed potential cytotoxic effect of compound 6 on MC38 cells at these concentrations, finding no significant effect on cell viability (Additional file 2: Figure S2). Taken together this indicated that compound 6 inhibited migration of the cells through the artificial ECM layer by blocking invasion as opposed to an anti-proliferative or cytotoxic effect.

To further evaluate the selectivity of compound 6, we decided to utilise an MC38 CTSS knock-down cell line, achieved through stable transfection of sh835 [6], to evaluate the effect versus a non-targeting control (NTC) shRNA. The sh835 CTSS knock-down line was validated by RT-PCR (Fig. 3b). The sh835 knock-down cells proved less invasive as anticipated versus the NTC cells (Fig. 3c). Furthermore, treatment of the NTC cells with compound 6 resulted in a reduced level of cell invasion comparable to the sh835 cells. Importantly, treatment of the sh835 cells with compound 6 demonstrated no additional reduction in the invasion, strongly indicating selectivity of compound 6 towards CTSS. MTT analysis indicated no effect of compound 6 on cell viability on either the MC38 NTC or sh835 cell lines (Additional file 3: Figure S3).

We have previously demonstrated in vitro that CTSS inhibition can attenuate endothelial tube formation, a process that underpins neoangiogenesis [36]. HUVEC cells were plated out on Matrigel coated plates and tube formation quantified after 18 h. Treatment of these cells with compound 6 elicited a mild but significant reduction in average tubule length at 100 nM (Fig. 3d). Taken together these in vitro studies demonstrate that compound 6 could inhibit CTSS to block pro-tumorigenic characteristics of tumor and endothelial cells, warranting further investigation with in vivo models.

### Evaluation of compound 6 in murine MC38 syngeneic model

**Fig. 4:**
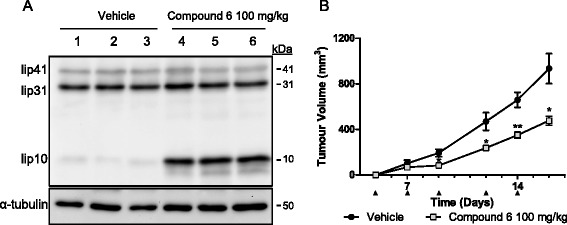
Compound 6 blocks CD74 degradation in murine spleen and reduces progression in MC38 tumor model. **a** C57BL/6 mice were treated with 100 mg/kg compound 6 for 18 h before harvesting of spleens and subsequent analysis of CTSS substrate lip10 by western blot. Results demonstrated compound 6 to block CTSS degradation of lip10 in murine spleen (3 mice/group). **b** Treatment of MC38 tumors syngeneic model with compound 6 (on days indicated by black arrow) significantly reduced tumor volume over 16 days (*P<0.05; **P<0.01)

To ensure that dosing of compound 6 in vivo would sufficiently block tumor CTSS, we first evaluated the ability of compound 6 to block lip10 degradation in C57BL/6 mice spleens upon administration. Mice were treated with compound 6 (100 mg/kg) and the level of CD74 degradation in their splenic lysates was evaluated. These experiments revealed a clear accumulation of lip10 fragment in mice (group of three) treated with compound 6 in comparison to mice treated with vehicle control (Fig. 4a). Extrapolation of band strength furthermore revealed lip10 band strength to be increased by ~13.13-fold, consistent with the effect observed in vitro using the Raji cells (Additional file 4: Figure S4).

We then examined the effect of compound 6 treatment on mice bearing MC38 syngeneic colorectal adenocarcinoma tumors. This model provides a fully immunocompetent microenvironment and we have used this model previously in our genetic ablation studies examining contribution of CTSS to tumor growth when derived from the tumor and/or the stoma [16]. We monitored MC38 tumor growth over a 16 day period with treatment of compound 6 or vehicle control delivered via intraperitoneal injection starting on day zero and following every 2 to 3 days. Dosing of compound 6 resulted in a reduction in tumor progression of approximately 50 % by day 16 when compared to vehicle-treated controls (Fig. 4b), an effect similar to what we had previously observed with CTSS depletion [16]. The weights of these animals were also monitored during the treatment timecourse, confirming no adverse effects of the inhibitor on mice weight and appearance (Additional file 5: Figure S5). During the course of this study, we also analysed serums from the tumor bearing animals and assessed CCL2 levels. Consistent with our previous report that CTSS inhibition can block CCL2 production [6], here we found a reduction in CCL2 levels at day 13, 24 h after the 5^th^ administration of compound 6 (Additional file 6: Figure S6).

### Evaluation of compound 6 on human MCF7 xenograft tumors

**Fig. 5:**
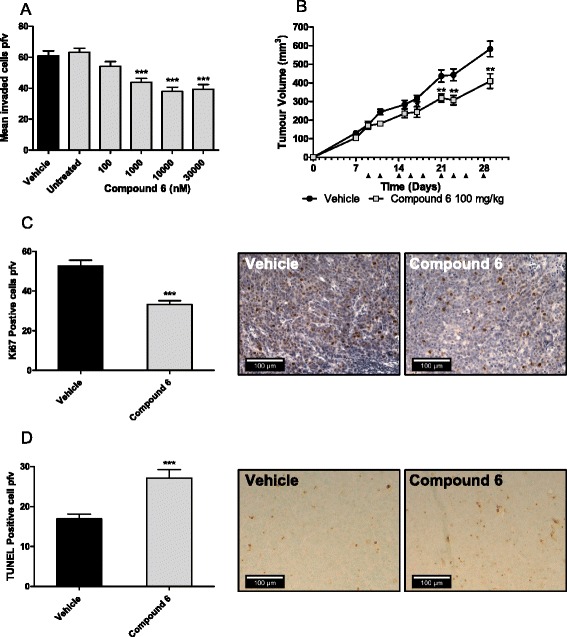
Compound 6 reduces MCF7 tumorigenesis in vitro and in vivo. **a** Compound 6 demonstrated a significant concentration dependent reduction in MCF7 invasion from 100 nM (***P<0.001). **b** MCF7 cells were propagated in BALB/c nude mice and subsequently treated with compound 6 (100 mg/kg) over the course of 29 days, indicated by black arrows. Treatment with compound 6 caused a significant reduction in tumor volume of 31 % versus vehicle by day 29 (**P<0.01). Immunohistochemical analysis of proliferation was carried out on MCF7 tumor sections. **c** Compound 6 caused a significant 0.62-fold reduction in tumor proliferation (***P<0.001), as demonstrated in the representative images. **d** TUNEL analysis of apoptosis was carried out on the MCF7 tumor sections. Compound 6 treatment demonstrated a 1.60-fold increase of apoptosis within the tumor sections (***P<0.001), as demonstrated in the representative images

Previously we have examined the inhibition of CTSS in human astrocytoma and colorectal tumors [24], but recently Sevenich and colleagues have shown that CTSS inhibition may also have utility in breast cancer [19]. MCF7 cells are oestrogen dependent adenocarcinomas that have previously been shown to express CTSS [37], and have previously been used as a model of tumor invasion in a number of other studies [38–41]. Taking these findings together, we examined whether the anti-tumor effects of compound 6 translated to a more human relevant model. Indeed, evaluation of compound 6 on MCF7 cell invasion demonstrated a dose dependent reduction in tumor cell invasion (Fig. 5a); which was independent of any effects on cell viability (Additional file 7: Figure S7).

The impact of compound 6 in vivo was then evaluated in the MCF7 xenograft model. The treatment of the mice resulted in a 30 % reduction of tumor growth by day 29 compared to vehicle-treated controls (Fig. 5b). To determine any affect on the health of the mice, their weights were obtained at the beginning of the study and treatment with compound 6 did not affect the health of the mice (Additional file 8: Figure S8).

Upon completion of the xenograft study, the mice were sacrificed, tumors harvested and MCF7 tissue sections prepared and stained for proliferation marker Ki67. Evaluation of Ki67 positive cells revealed that cell proliferation was significantly reduced in the compound 6 treatment group (Fig. 5c). Evaluation of compound 6 effect on neo-angiogenesis was also carried out by CD34 staining, however, no observable differences could be confirmed between mice treated with compound 6 and control mice (Data not shown). Finally, the presence of apoptotic cells was evaluated using TUNEL staining. The results demonstrated an increased level of apoptosis in the compound 6 treated MCF7 tissue sections when compared with vehicle-treated controls (Fig. 5d). Taken together, CTSS inhibition was shown to block tumor progression, through decreased invasion, decreased proliferation and increased apoptosis, consistent with previous observations [16].

## Discussion

In this report we have described the validation and characterisation of small molecule CTSS inhibitor, compound 6 using activity, cellular and murine tumor models. We validated selectivity of compound 6 towards CTSS using a recombinant enzyme activity screening before confirming its effect on cellular activity. Compound 6 demonstrated the ability to block both murine and human cell invasion and to disrupt endothelial tube formation showing its activity as an anti-angiogenic compound. Selectivity of compound 6 towards CTSS in the cellular environment was confirmed using an MC38 knock-down model, whereby treatment of CTSS depleted cells with compound 6 produced no further reduction in invasion. The efficacy of compound 6 was validated in vivo, first demonstrating inhibition of murine spleen CTSS and subsequently using MC38 syngeneic and MCF7 xenograft tumor models, where compound 6 treatment demonstrated reduced tumor volume, complemented by reduced cell proliferation and concomitant increase in apoptosis.

Over the past 15 years, there has been an increasing body of evidence reporting increased expression of CTSS in a variety of malignancies [42–44], with this increased expression being associated with poorer prognosis in grade IV astrocytomas and colorectal carcinomas [13, 14]. Genetic ablation studies targeting CTSS has demonstrated reduced tumor progression in murine models [15, 16]. Indeed, CTSS has been shown to capable of degrading a number of substrates including ECM proteins, pro-and anti-angiogenic substrates, proteins involved in metastases and pro-inflammatory cytokines/chemokines [5, 6, 17–19].

Compound 6 was previously reported to be a potent and selective CTSS inhibitor [26]. Generation of *K*_i_ values for compound 6 versus CTSS and family members K, L and B (Additional file 1: Figure S1) were consistent with IC_50_ values previously reported [26]. Importantly, this inhibitor demonstrated membrane permeability and cross species reactivity by blocking lip10 degradation both in vitro using Raji cells and subsequently in vivo in C57BL/6 spleen tissue [27] and indicating suitability of compound 6 as a tool compound to aid the elucidation of CTSS in subsequent experiments. When applied in both MC38 and MCF7 transwell invasion assays, compound 6 demonstrated the ability to reduce tumor cell invasiveness in a manner consistent with pharmacologically blocking CTSS activity [24].

The inhibitor used here has previously been utilised in vivo in *Apoe*^-/-^ mice, observing the effect of CTSS in lung granuloma, atherosclerotic lesions [27, 28] and in a murine asthma model [45]. Here, the effectiveness of compound 6 in vivo was validated in murine cancer models for the first time. The role of CTSS in colorectal tumors has been well elucidated in patients, with high expression indicating poorer prognosis [14]. A dual depletion MC38 model has previously been established, whereby both host *and* tumor CTSS has been depleted, demonstrating a reduction in tumor progression and growth [16]. Consistent with this, compound 6 demonstrated a reduction in tumor volume using a matching MC38 synergistic tumor model. Interestingly, a greater reduction in tumor volume was observed in the MC38 model than the MCF7 model. It is possible that this may be a consequence of harbouring the tumors in immunocompromised BALB/c mice, which are lacking some stroma-mediated interactions [46, 47].

Recently, a role for CTSS has been uncovered with respect breast-to-brain metastasis. Inhibition using a CTSS inhibitor VBY-999, reduced CTSS mediated metastases in an MDA-MB-231 triple negative breast cancer model [19]. Interestingly, in contrast to our own observations using a luminal A MCF7 model, no reduction in tumor burden was observed with respect the MDA-MB-231 xenograft and VBY-999 treatment [19], suggesting that classical CTSS pathological roles in ECM degradation and neo-angiogenesis may be subtype specific, warranting a more in-depth analysis of breast cancer heterogeneity at the patient sample level.

Compound 6 demonstrated only a mild anti-angiogenic effect in vitro against HUVEC tube formation. Interestingly, this effect was not as pronounced as previously observed with FSN0503 [24]. CTSS has been shown to play a key role in neo-vascularisation at the tumor site [24, 34, 48, 49]. Previously, depletion of CTSS in vivo has also demonstrated a reduction in angiogenesis using an MC38 syngeneic model [16]. Analysis of harvested MCF7 tumors using CD34 vascular marker did not demonstrate a difference between groups (data not shown). This is perhaps unsurprising due to the smaller effect exhibited by compound 6 on HUVEC tube formation in comparison to FSN0503. As a result, further analysis, possibly using a cell line that exhibits higher levels of vascularisation than these MCF7 tumors, may reveal further anti-tumorigenic effects.

## Conclusions

In conclusion, small molecule inhibitor compound 6 demonstrates the ability to block tumor progression both in vitro and in vivo. Our results compliment previous observations in colorectal carcinomas and furthermore, have demonstrated for the first time, blockade of tumor progression in a breast cancer murine model. The reduction in tumor progression caused by CTSS inhibition necessitates further analysis of expression in human clinical samples, to identify patients who would benefit from CTSS inhibition. Indeed, in the past chemotherapeutic and radiation treatment has resulted in increased expression of CTSS likely due to cellular stress [22, 37, 50], suggesting inhibition of CTSS may be crucial for patient outcome. We hypothesize, that the development of small molecule CTSS inhibitor compound 6 will facilitate the understanding of mechanistic roles for CTSS in disease and furthermore, facilitate its characterisation in cancers which are currently poorly defined.

## Additional files

Additional file 1: Figure S1. Table of compound 6 *K*_i_ values (3 s.f.) versus recombinant cathepsin S, K, V, L and B. (PPTX 36 kb) Additional file 2: Figure S2. MTT assay shows Compound 6 not to affect MC38 cell viability at 100 or 1000 nM. (PPTX 64 kb) Additional file 3: Figure S3. MTT assay shows addition of sh835 CTSS knock-down construct and/or treatment with compound 6 at 1000 nM does not MC38 affect cell viability. (PPTX 56 kb) Additional file 4: Figure S4. Extrapolation of lip10 band strength using densitometry reveals a 13.13-fold increase in mice treated with compound 6 versus controls. (PPTX 42 kb) Additional file 5: Figure S5. Compound 6 treatment regime did not affect mouse weights during the MC38 syngeneic study, suggesting tolerance. (PPTX 62 kb) Additional file 6: Figure S6. Analysis of MC38 tumor bearing mice serum at day 13 (24 h after compound 6 administration) revealed mild reduction in CCL2 levels. (PPTX 51 kb) Additional file 7: Figure S7. MTT assay shows compound 6 does not affect MCF7 cell viability at 100-30000 nM. (PPTX 62 kb) Additional file 8: Figure S8. Compound 6 treatment regime did not affect mouse weights during the MCF7 xenograft study, suggesting tolerance. (PPTX 60 kb)

## Abbreviations

CTSS: Cathepsin S
ECM: Extracellular matrix
TAM: Tumor associated macrophage
CD74: Invariant chain
lip10: 10 kDa invariant chain fragment
NTC: Non-targeting control
