# Supplementary figures and images for: A bioavailable cathepsin S nitrile inhibitor abrogates tumor development
Source: Mol Cancer. 2016 Apr 21;15:29. doi: 10.1186/s12943-016-0513-7 (PMC4839156; doi:10.1186/s12943-016-0513-7)

## Slide 1
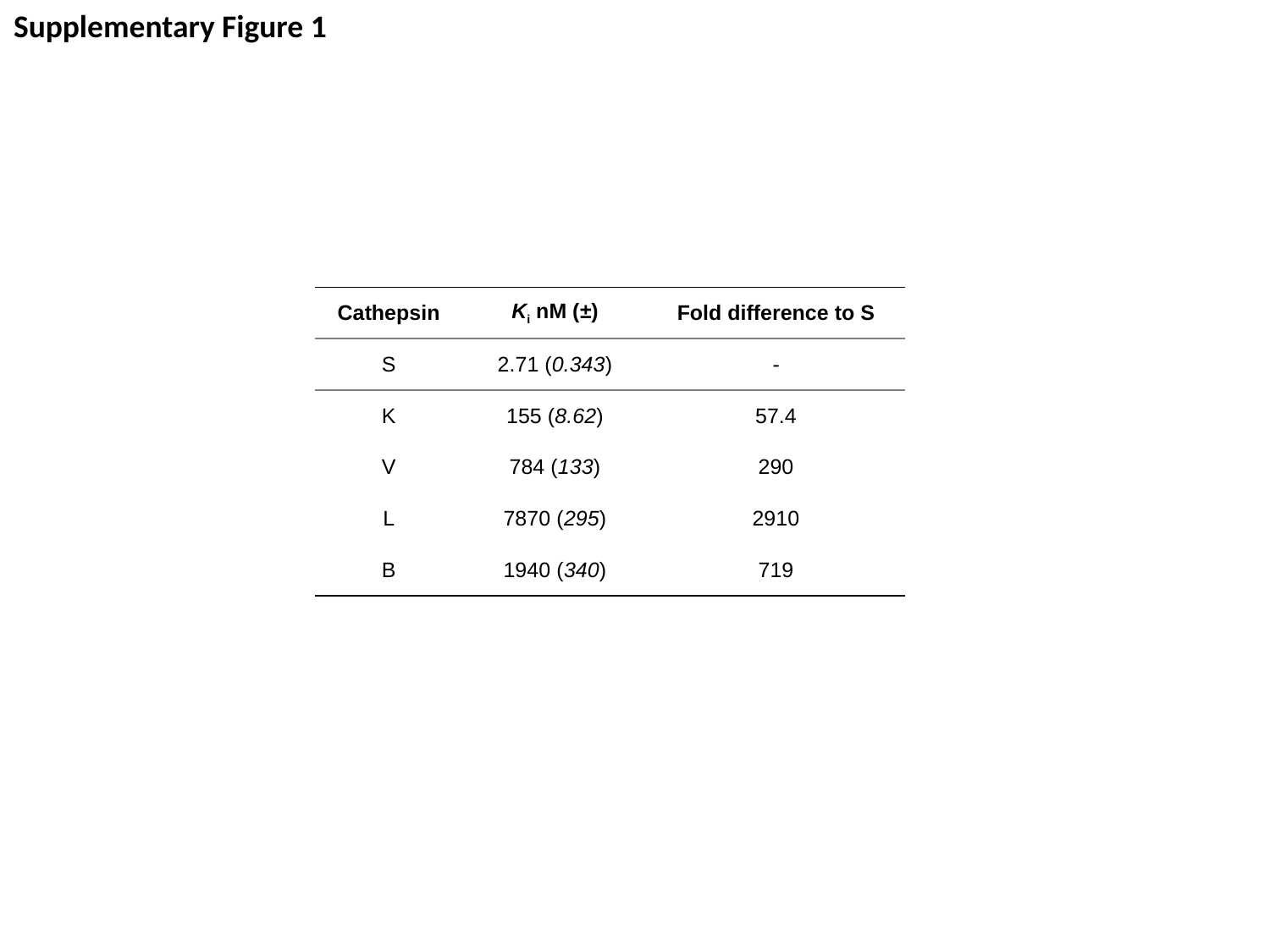

Supplementary Figure 1
| Cathepsin | Ki nM (±) | Fold difference to S |
| --- | --- | --- |
| S | 2.71 (0.343) | - |
| K | 155 (8.62) | 57.4 |
| V | 784 (133) | 290 |
| L | 7870 (295) | 2910 |
| B | 1940 (340) | 719 |

Supplement: Additional file 1: Figure S1. — Table of compound 6 K i values (3 s.f.) versus recombinant cathepsin S, K, V, L and B. (PPTX 36 kb) [file 12943_2016_513_MOESM1_ESM.pptx]

## Slide 1
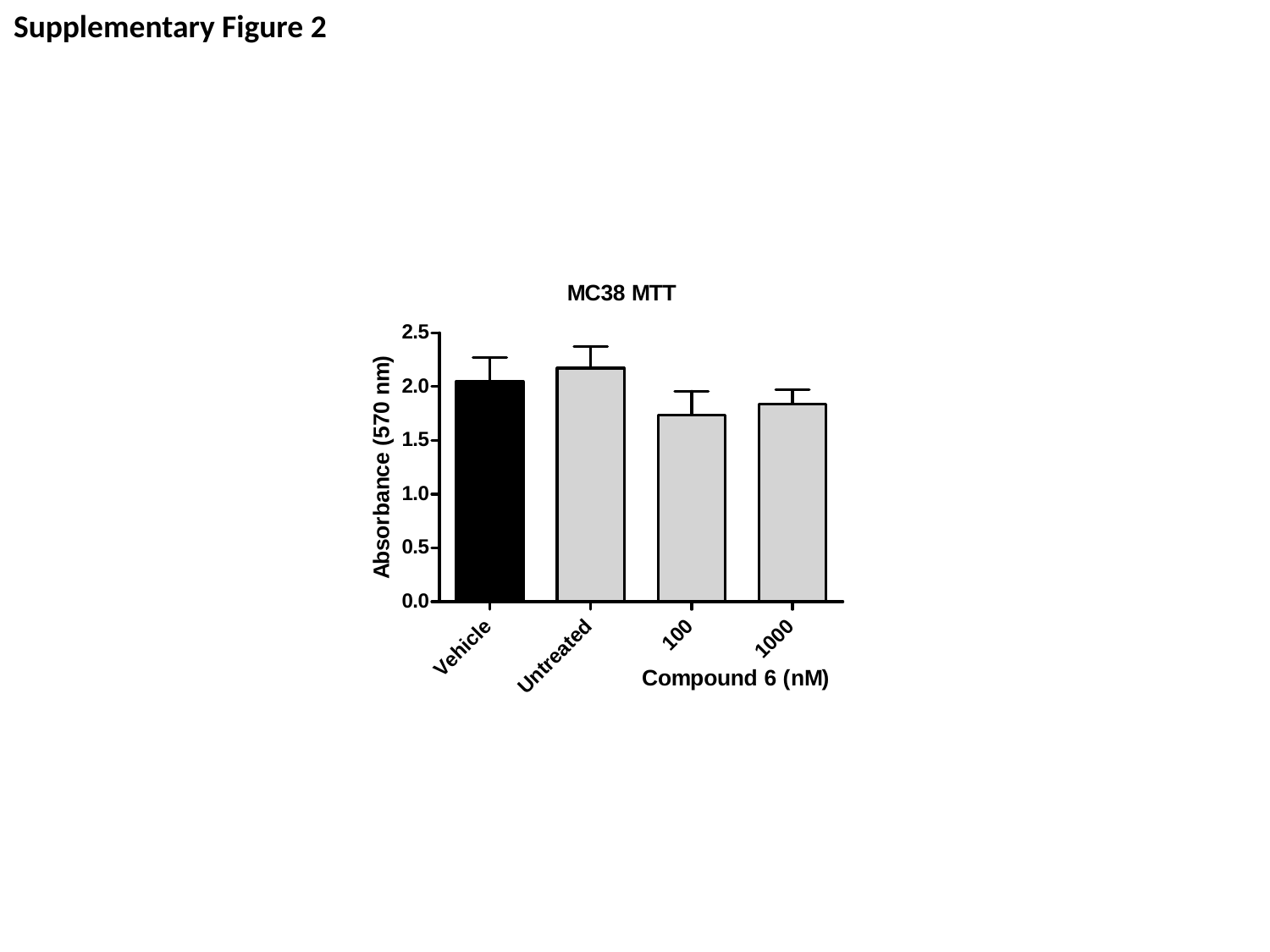

Supplementary Figure 2

Supplement: Additional file 2: Figure S2. — MTT assay shows Compound 6 not to affect MC38 cell viability at 100 or 1000 nM. (PPTX 64 kb) [file 12943_2016_513_MOESM2_ESM.pptx]

## Slide 1
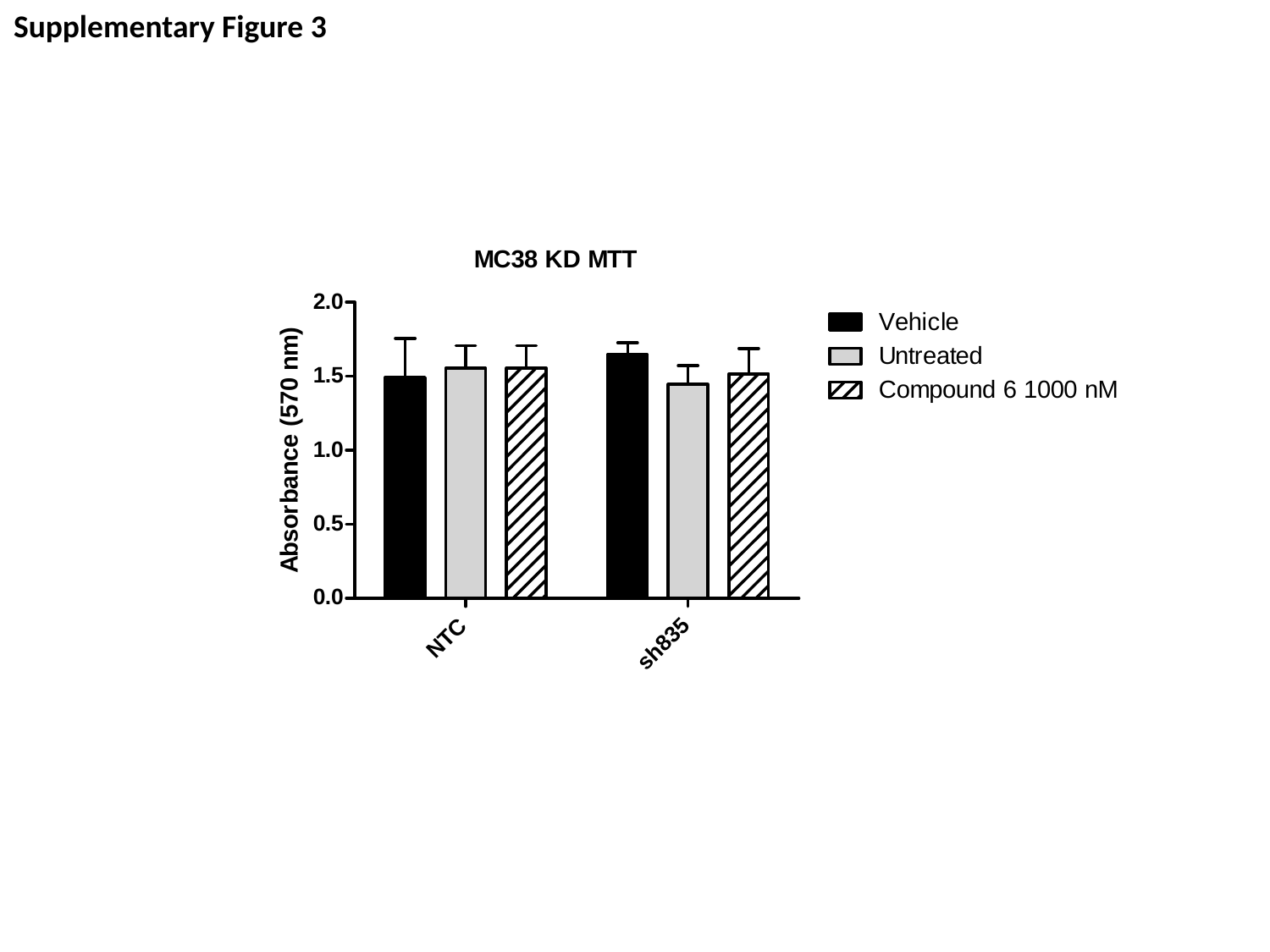

Supplementary Figure 3

Supplement: Additional file 3: Figure S3. — MTT assay shows addition of sh835 CTSS knock-down construct and/or treatment with compound 6 at 1000 nM does not MC38 affect cell viability. (PPTX 56 kb) [file 12943_2016_513_MOESM3_ESM.pptx]

## Slide 1
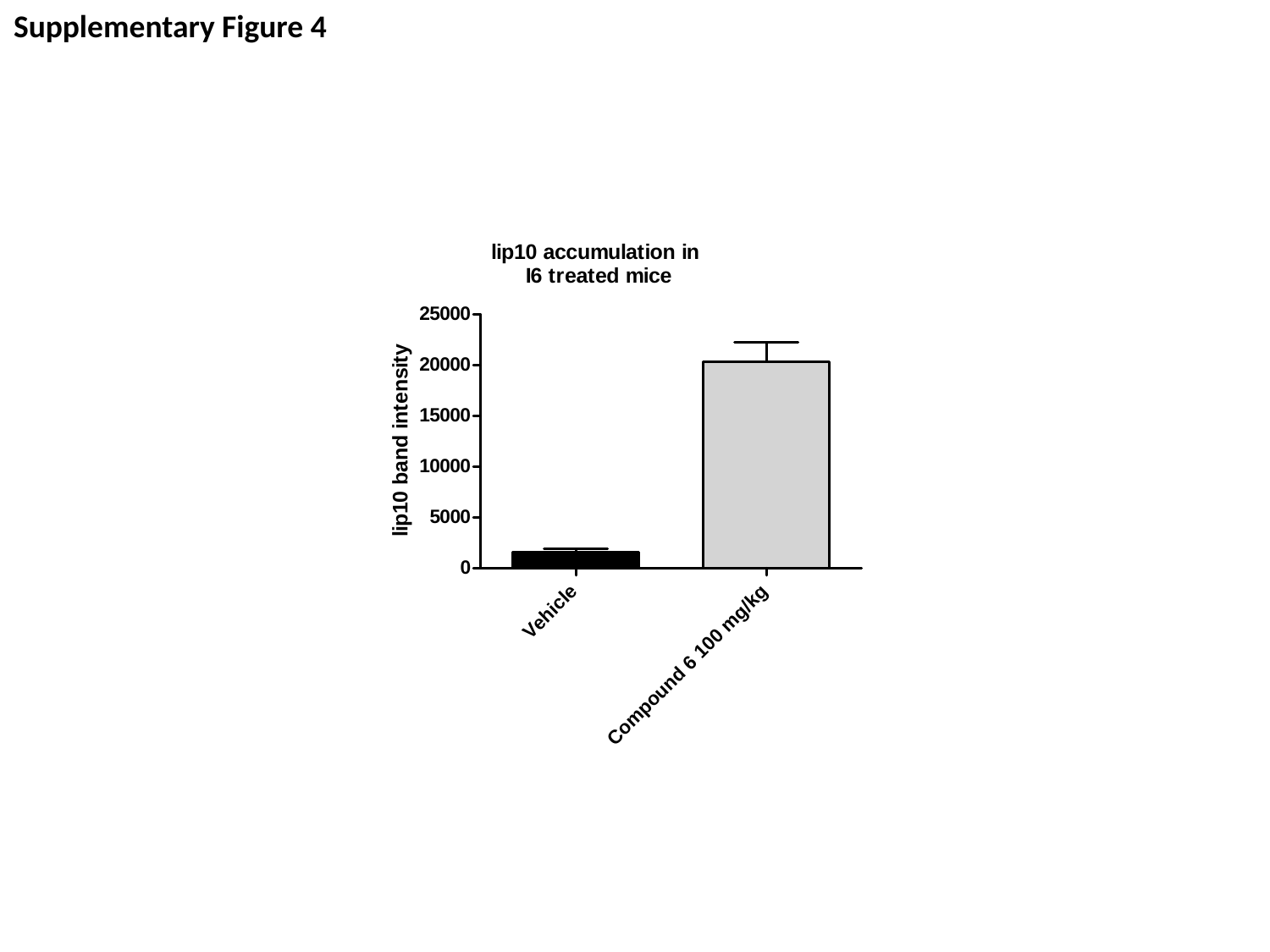

Supplementary Figure 4

Supplement: Additional file 4: Figure S4. — Extrapolation of lip10 band strength using densitometry reveals a 13.13-fold increase in mice treated with compound 6 versus controls. (PPTX 42 kb) [file 12943_2016_513_MOESM4_ESM.pptx]

## Slide 1
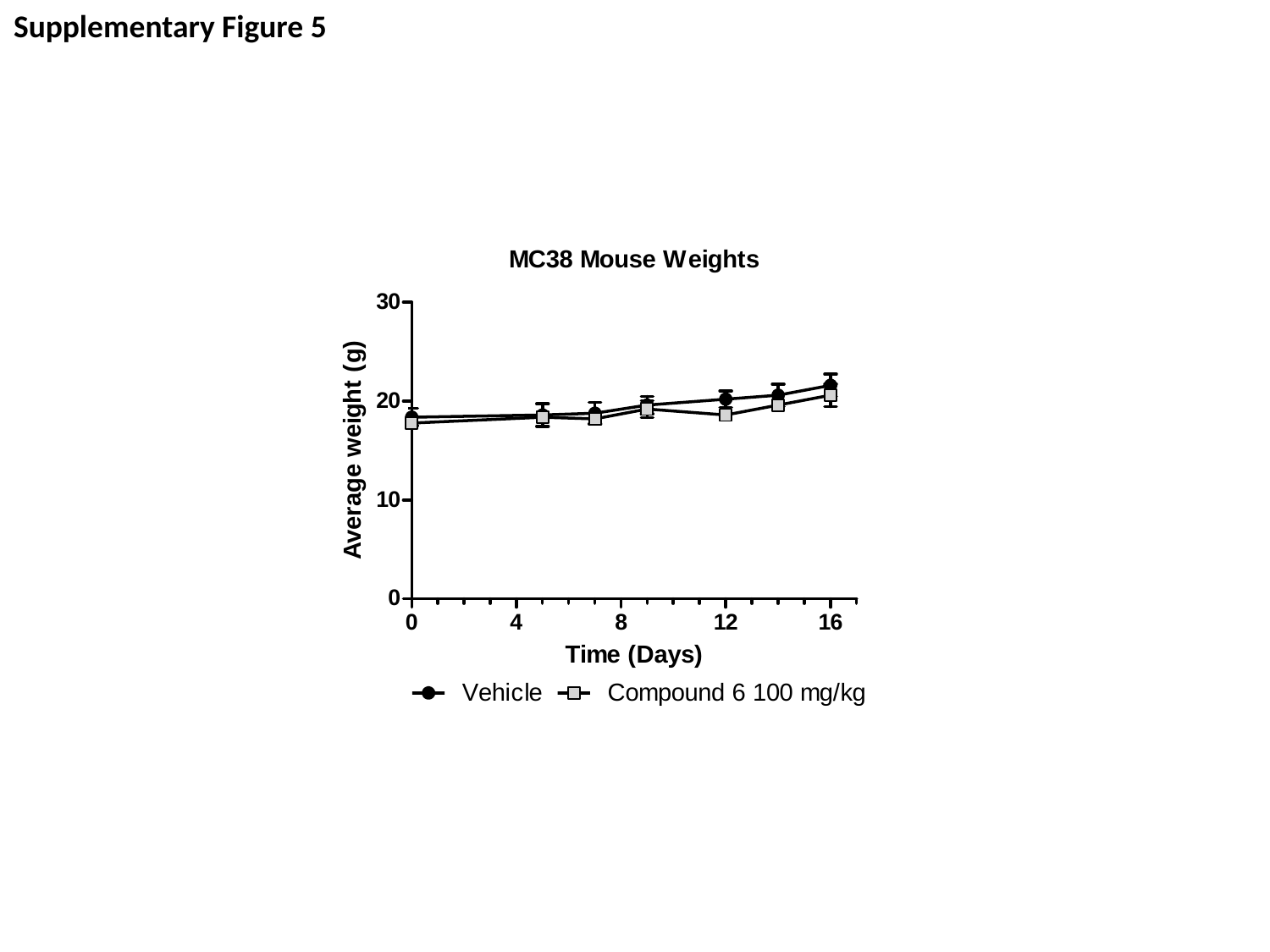

Supplementary Figure 5

Supplement: Additional file 5: Figure S5. — Compound 6 treatment regime did not affect mouse weights during the MC38 syngeneic study, suggesting tolerance. (PPTX 62 kb) [file 12943_2016_513_MOESM5_ESM.pptx]

## Slide 1
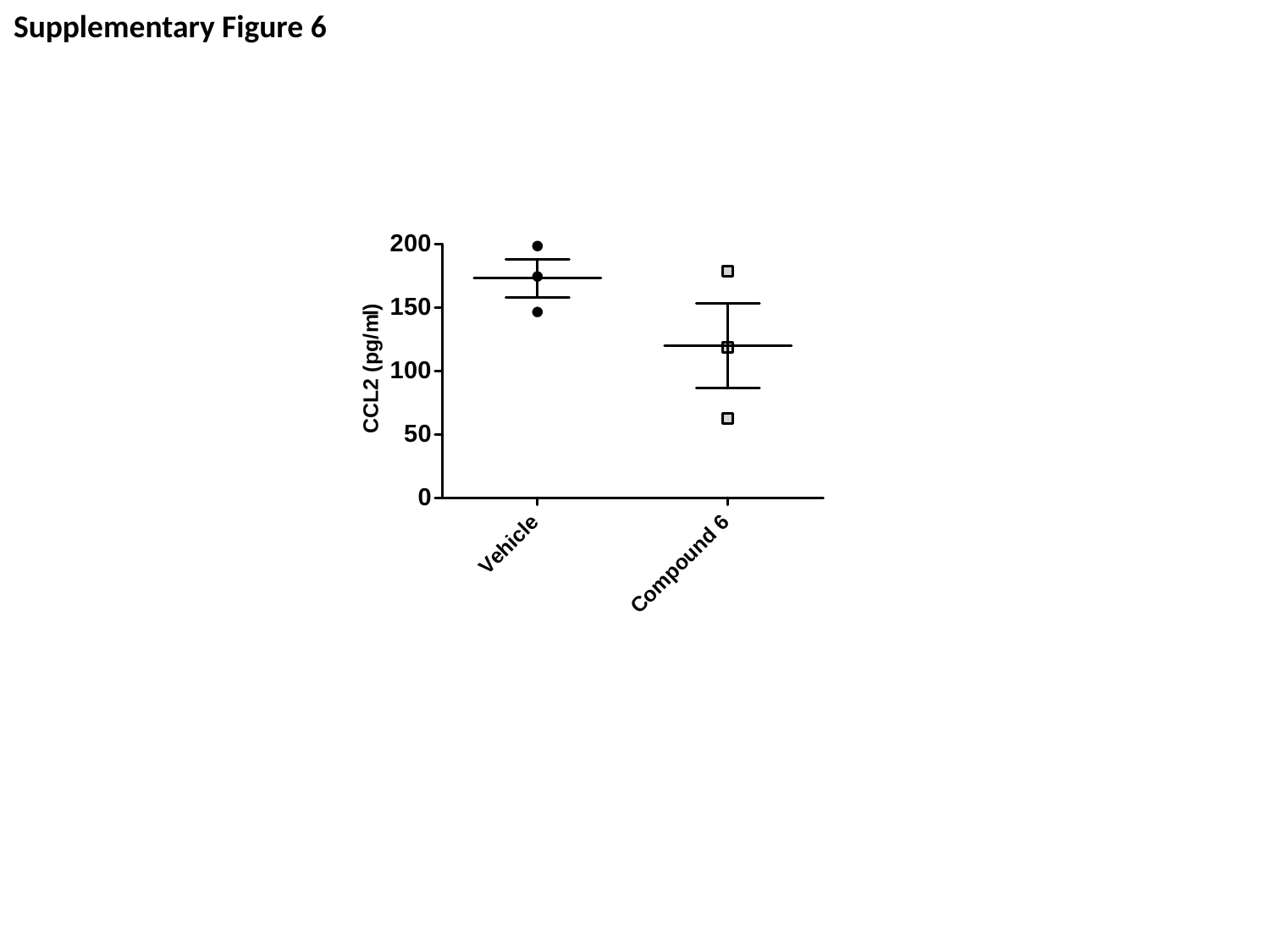

Supplementary Figure 6

Supplement: Additional file 6: Figure S6. — Analysis of MC38 tumor bearing mice serum at day 13 (24 h after compound 6 administration) revealed mild reduction in CCL2 levels. (PPTX 51 kb) [file 12943_2016_513_MOESM6_ESM.pptx]

## Slide 1
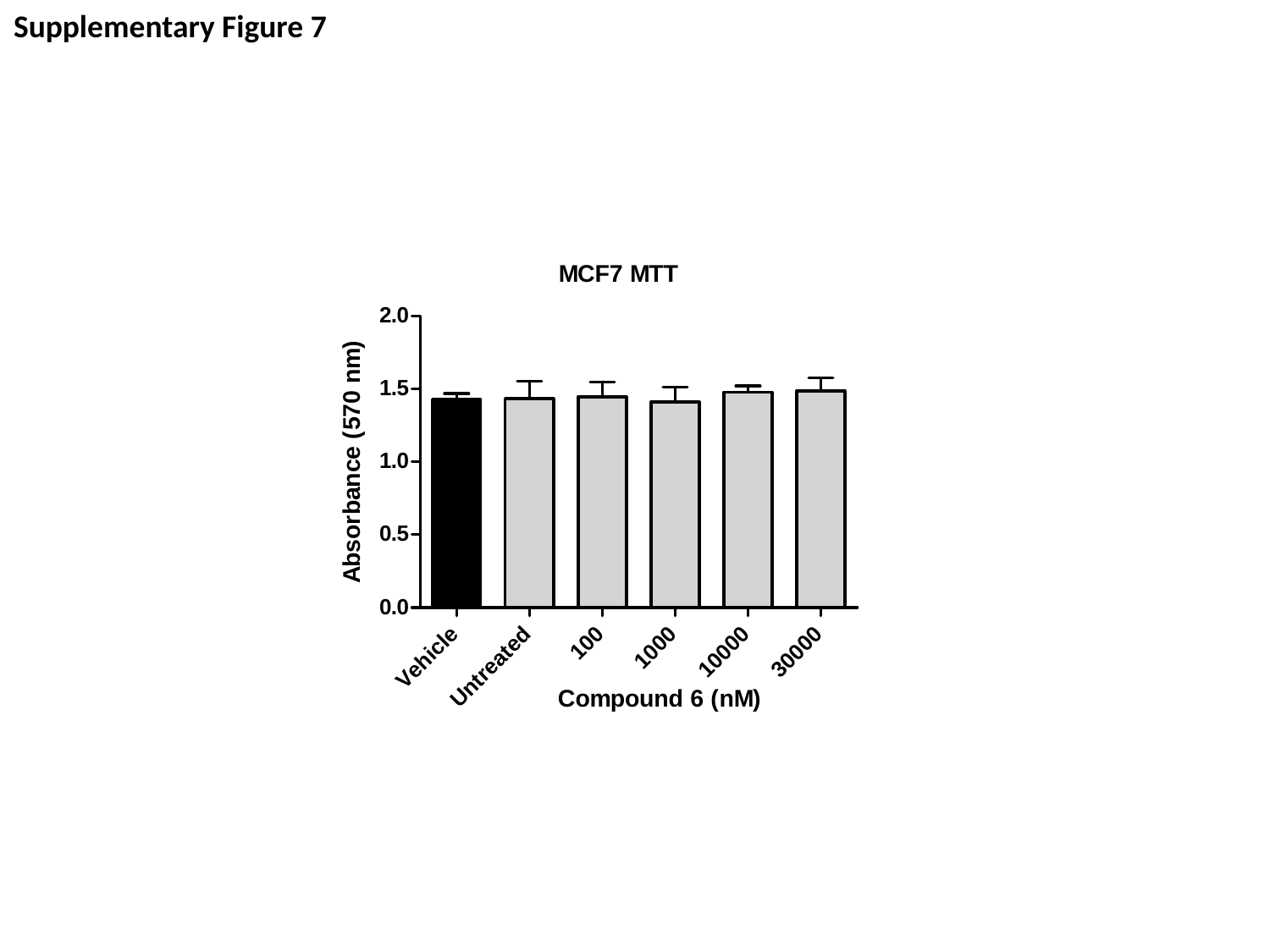

Supplementary Figure 7

Supplement: Additional file 7: Figure S7. — MTT assay shows compound 6 does not affect MCF7 cell viability at 100-30000 nM. (PPTX 62 kb) [file 12943_2016_513_MOESM7_ESM.pptx]

## Slide 1
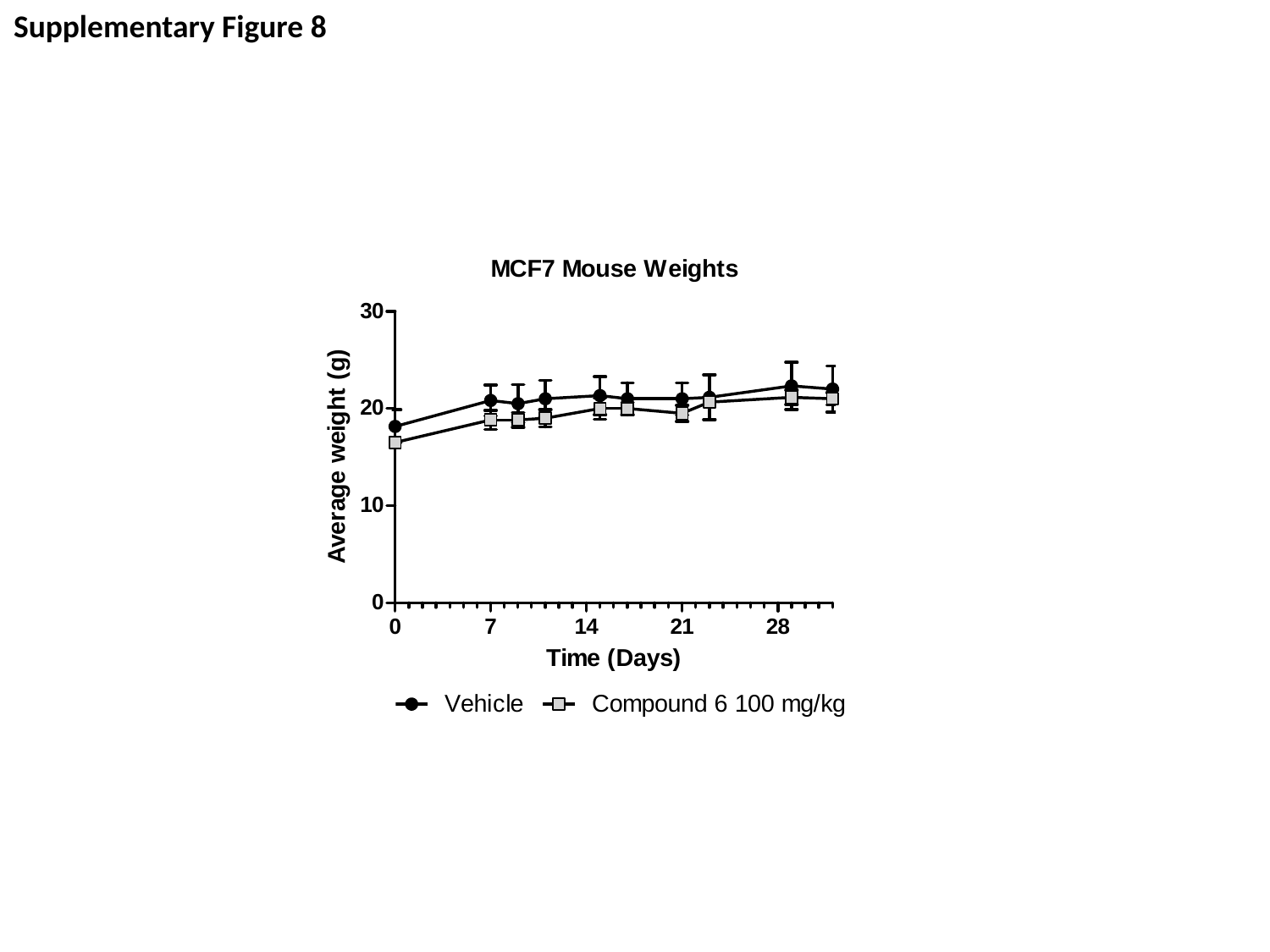

Supplementary Figure 8

Supplement: Additional file 8: Figure S8. — Compound 6 treatment regime did not affect mouse weights during the MCF7 xenograft study, suggesting tolerance. (PPTX 60 kb) [file 12943_2016_513_MOESM8_ESM.pptx]
